# Thanks to our reviewers of 2019 and call to review in 2020!

**DOI:** 10.1080/13814788.2020.1714310

**Published:** 2020-02-05

**Authors:** Jelle Stoffers

**Affiliations:** (Editor-in-Chief)

The Editors of the *European Journal of General Practice* (EJGP) would like to express their gratitude to all reviewers who have advised us during the year 2019. To acknowledge their essential contribution to the European Journal of General Practice, we have listed the names of all referees who completed at least one review report in 2019.

Aarendonk, Diederik; Abecain, Francisco; Adab, Peymane; Adam, Rosalind; Adams, Margo; Adriaenssens, Niels; Afonso, Luis; Agyeman-Yeboah, Joana; Akman, Mehmet; Albasri, Ali; Alvarez, Isabel; Anthierens, Sibyl; Aragonés, Enric; Archibald, Lennox K.; Augustine, Erin M.; Avinadav, Efrat; Bak, Marieke; Bayona, Carol; Begum, Aysha; Belbasis, Lazaros; Berenguera, Anna; Best, James D.; Bethell, Christina D.; Bickel, Horst; Ble, Alessandro; Blozik, Eva; Boeckxstaens, Pauline; Bøgelund, Mette; Boogaard, Jannie A.; Borrell, Francisco; Bosmans, Jan; Brandenbarg, Daan; Brandt, C.; Brayne, Carol; Broekema, Susanne; Brown, Stepahnie; Buchanan, Jo; Bucher, Heiner C.; Bullock, Laurna; Buntinx, Frank; Busschers, Wim; Cabral, Christie; Cals, Jochen; Campbell, Paul; Campiñez, Manuel; Carbonnel, François; Carrasquillo, Olveen; Cartwright, Claire; Chen, Meixuan; Chung, Paul J.; Clark, Christopher E.; Clark-Grill, Monika; Cohen, Debbie L.; Cohen, Jordana; Coker, Tumaini R.; Colliers, Annelies; Cooper, Jason; Cordoba, Gloria Correia de Sousa, Jaime; Cots, Josep Maria; Cottrell, Lizzie; Cutler, Lizl Dal Bianco, Peter Davis, Ann de la Flor, Josep; De Smedt, Delphine; de Sutter, An; Deasy, C.; Denekens; Joke; Digby, Robin; Dijkland, Simone; Dinant, Geert-Jan; Dinnes, Jacqueline; Discigil; Guzel; Doherty, Sarah; Dreno, Brigitte; Duggleby, Wendy; Duimel-Peeters, Inge; Bell, Mark H.; Eichler, Tilly; Eldin, Carole; Elfström, K.Miriam; Ellbrant, Julia A.; Endacott, Ruth; Falger, Paul; Fasset, Rob; Fernandes, Susana; Finney, Andrew; Firet, Lotte; Fischer, Gabriele; Fletcher, Benjamin; Fouche, Pieter; Fox, Larry A.; Freund, Tobias; Gallagher, Joe; Geersing, Geert-Jan; Gerber, Jeffrey; Gertig, Dorota M.; Godycki-Cwirko, Maciek; Goicolea, Isabel; Goodloe, Jeffrey; Grand, Johannes; Grassi, Guido; Greer, Joy A.; Griebling, Thomas; Griffin, Tania; Gross, D.; Gryglewska, Barbara; Gucek, Nena; Guedj, Romain; Gunvar, Tolga; Guenoer, Ken; Haidinger, Gerald; Hallberg, Ingalill Rahm; Hamashima, Chisato; Hansmann, Yves; Harskamp, Ralf; Hay, Alastair; Hayward, Gail Nicola; Hedin, Katarina; Helliwell, Toby; Helsper, Charles; Hernandez Santiago; Virginia; Heron, Neil; Herrmann, Wolfram; Hinrichs, Timo; Holm, Maja; Hopstaken, Rogier; Huesing, Paul; Hughes, Lloyd; Huibers, Linda; Iacob, Mihai; Ingram, Jenny; Janne Fangel, Jensen; Jardim, Thiago Veiga; Jensen, Henry; Jensen, Martin; Jeyashree, Kathiresan; Junius-Walker, Ulrike; Kanabar, Dipak; Keiren, Suzanne; Kendir, Candar; Keohane, David; Kern, Jean-Baptiste; Kluetz, Paul; Knottnerus, Bart; Koes, Bart; Koshiaris, Constantinos; Kostev, Karel; Kotseva, Kornelia; Kraaijvanger, Nicole; Krist, Alex H.; Kuşkonmaz, Şerife; Lagro-Jansen, Toine; Lamarre-Cliche; Maxime; Lan, Patrick G.; LeBlanc, Constance; Leeflang, Mariska; Legido-Quigley, Helena; Legrand, Deplphine; Leysen, Bert; Lieb, Klaus; Liira, Helena; Lim, Anita; Lischka, Martin; Llor, Carl; Lloyd-Williams, Mari; Loehr, Laura; Lofters, Aisha; Lowres, Nicole; Maarsingh, Otto; Madurell, Jordi; Maertens, H.; Maerz, Richard; Magin, Parker; Mahabee-Gittens, E. Melinda; Mahajan, Preetam; Maier, Manfred; Mallen, Christian; Marshall, Sara; Martin Lesende, Iñaki; Martinez-Gonzalez, Nahara; Marzuillo, Pierluigi; Mathes, Erin F. D.; Mathhys, Jan; Mazaheri, Monir; McCabe, Marita; McGorrian, Catherine; McManus, Richard J.; McWhannell, Nicola; Melbye, Hasse; Merianos, Ashley L.; Missiou, Aristea; Moberg, Anna; Moragas-Moreno, Ana; Morato, M. Lluisa; Morita; Tatsuya; Moser, Albine; Moth, Grete; Murphy, Marie; Muth, Christiane; Naouri, Diane; Nekhliyudov, Larrissa; Neri, Emanuele; Ntzani, Evangelia; Nygaard, Peter; O’Caoimh, R.; Olsman, Erik; Onakpoya, Igho; Pallan, Miranda; Park, Young-Jae; Parry, Emma; Perez Cachafeiro, Santiago; Peteiro, Jesus; Petek Ster, Marija; Petek, Davorina; Polomeni, Pierre; Prasad, Ramakrishna; Pujol, Jesus; Qaseem, Amir; Raman, Gowri; Rat, Cédric; Rattray, Nicholas A.; Rebnord, Ingrid; Reid, Jim; Rimoin, Anne; Roodbol; Petrie; Rouge-Bugat, Marie-Eve; Rubio Pérez, Francisco; Rubio-Guerra, Alberto; Rustoen, Tone; Sachs, Alfred; Sancho-Garnier, Helene; Schmidt, Morten; Schuers, Matthieu; Schwartz, Claire; Scolnik, Dennis; San Sebastian, Miguel; Senan Sanz, M. Rosa; Shepherd, Tom; Shewade, Hemant Deepak; Silina, Vija; Siontis, George; Siontis; Konstantinos; Spooner, Sharon; Staessen, Jan A.; Standage, Martyn; Stewart, Robert; Stoffers, Jelle; Sturman, N; Sultana, Farhana; Svab, Igor; Szecsenyi, Joachim; Tatsioni, Athína; Taylor, Elizabeth Johnston; Taylor, Leslie; Thompson, Paul; Thomsen, Janus; Thulesius, Hans; Thyrian, Jochen Rene; Tiro, Jasmin A.; Tomasik, Tomasz; Tonkin-Crine, Sarah; Topsever, Pinar; Trolle, Laura; Troncoso, Amelia; Twigg, Diane E.; Valderas, Jose M; van der Steen, Jenny T.; van Doorn, Sander; van Lieshout, Jan; van Marwijk, Harm; Van Royen, Paul; van Schayck, Constant; Vankrunkelsven, Patrick; Varva, Olimpia-Maria; Vaughan Sarrazin, Mary; Vázquez-López, Esther; Verbakel, Jan; Verhoeven, Veronique; Visca, Modesta; Vorspan, Florence; Wallace, Paul; Wancata, Johannes; Watzke, Herbert; van Weert, Henk; Weinzimer, Stuart A.; Wensing, Michel; Whited, John D.; Windak, Adam; Winer, Rachel L.; Wong, Eliza; Wu, Ann Chen; Wucherer, Diana; Young, Richard; Yuguero Torres, Oriol; Zhu, Dingliang; Zittleman, Linda; Zwakhalen, Sandra MG.

We want to thank you all for your appreciated assistance! Your review reports were very valuable feedback to the authors and helped us to make our editorial decisions. Currently, the *European Journal of General Practice* accepts around 20% of all submitted manuscripts.

Although our database of authors and reviewers steadily increases, the Editors do not always find it easy to find referees within the journal’s deadlines. Therefore, we sincerely hope that all reviewers want to continue their review work in the future. If you would like to update your reviewer account, e.g. to specify your expertise, please visit http://mc.manuscript-central.com/ejgp and log in to modify your profile.

In addition, we invite all readers of the *European Journal of General Practice* who would like to review manuscripts for this journal, to visit http://mc.manuscriptcentral.com/ejgp and to register as ‘new user’, i.e. to complete the details of their expertise. We provide a format to facilitate the review.

If you have comments regarding the journal or its peer review process, we invite you to contact the editorial office (Ms. Anneke Germeraad-Uriot, Editorial Assistant: ejgp-agermeraad@maastrichtuniversity.nl).

Jelle Stoffers On behalf of the Editorial Board of the *European Journal of General Practice*: Carl Llor, Manfred Maier, Christian Mallen, An de Sutter, Athina Tatsioni, Henk van Weert, Adam Windak, Anneke Germeraad-Uriot Jelle Stoffers (Editor-in-Chief) ejgp-jstoffers@maastrichtuniversity.nl

